# Supplementary figures and images for: Genome Sizes and the Benford Distribution
Source: PLoS One. 2012 May 18;7(5):e36624. doi: 10.1371/journal.pone.0036624 (PMC3356352; doi:10.1371/journal.pone.0036624)

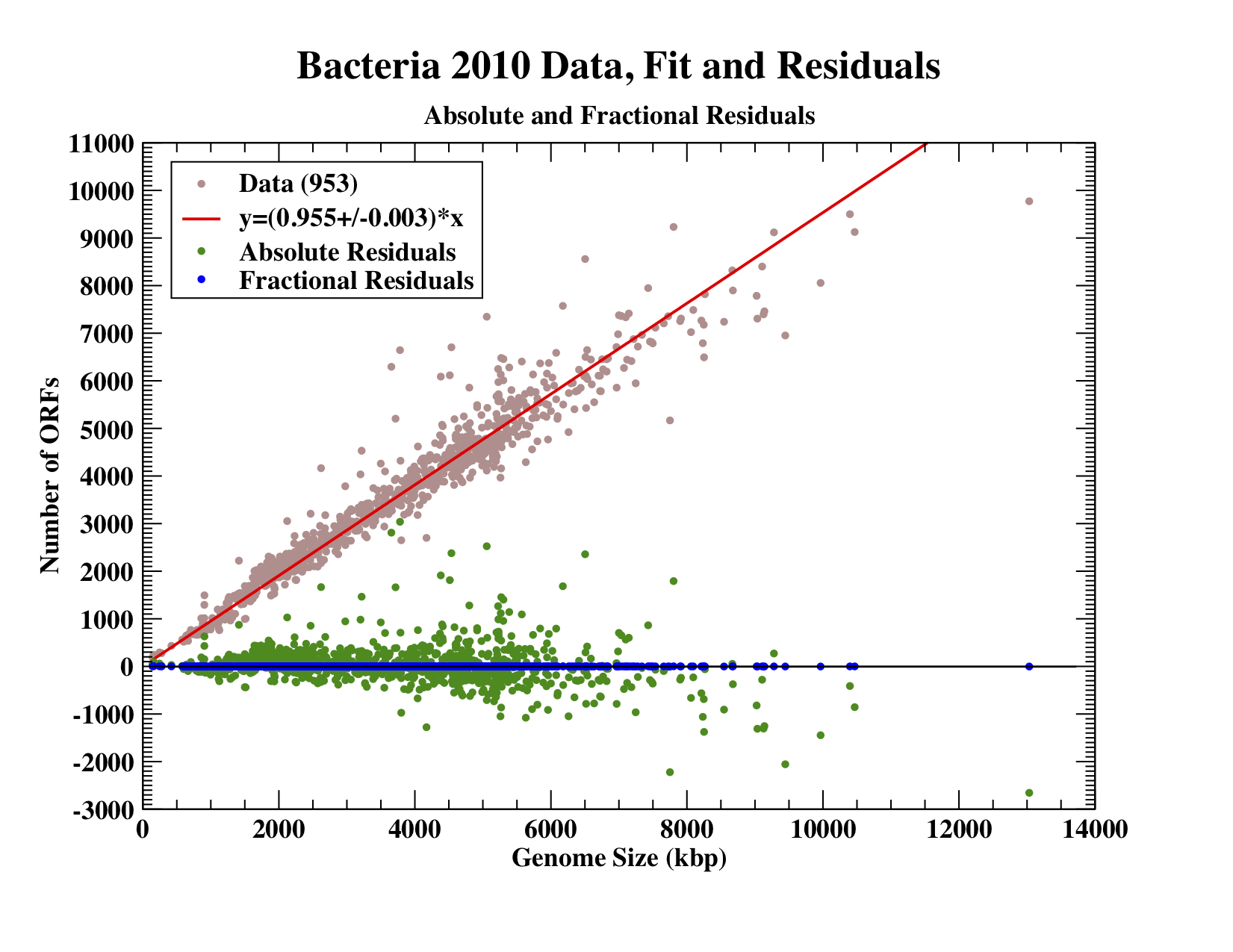

Supplement: Figure S1 — Bacteria Data, Fits and Residuals. Distribution of Bacteria data about the fit line (in red) and absolute residuals relative to that line (in green) as a function of genome size in kbp. Fractional residuals are shown in blue. (TIFF) [file pone.0036624.s001.tiff]

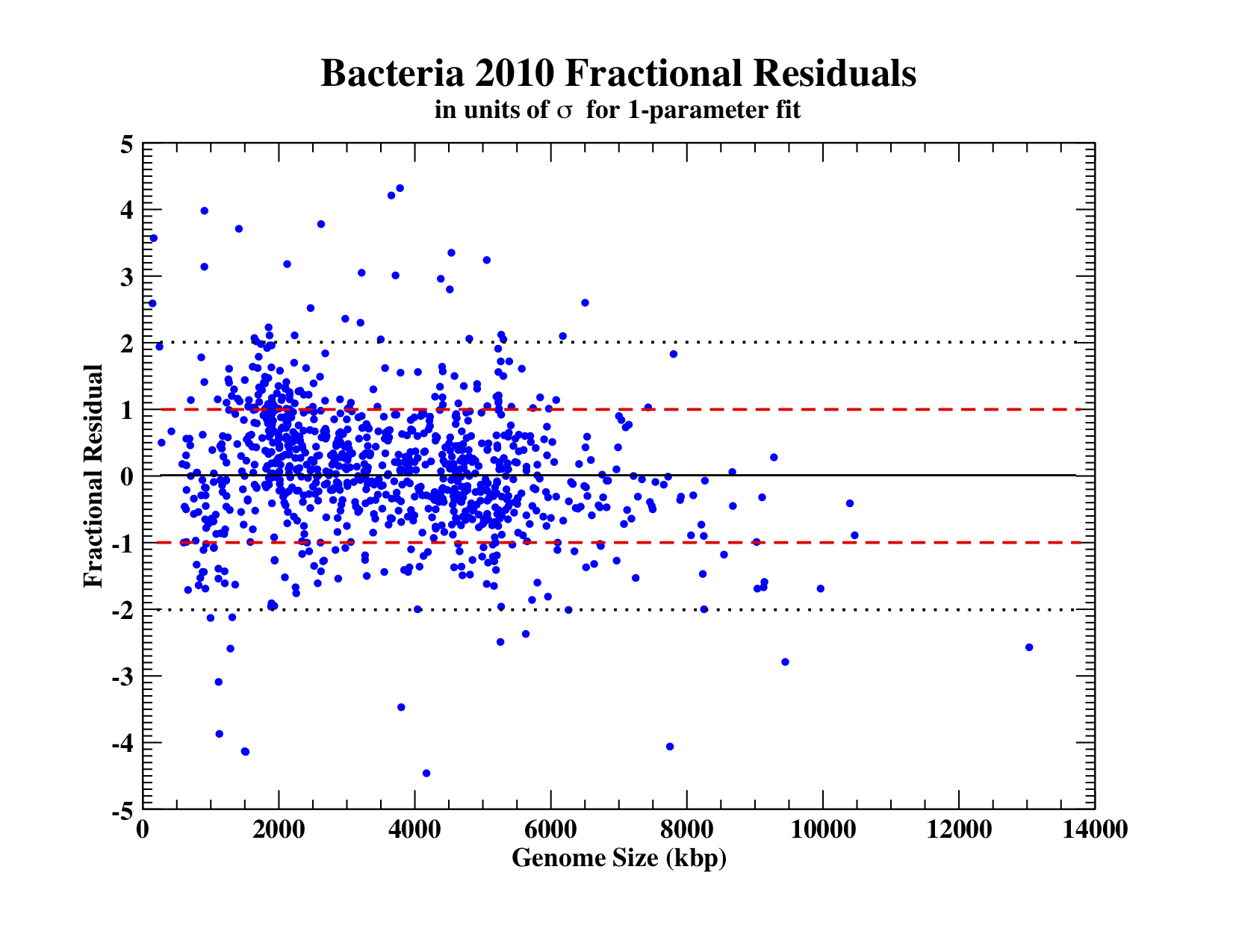

Supplement: Figure S2 — Bacteria Fractional Residuals. Distribution of Bacteria fractional residuals (in blue) as a function of genome size in kbp. The black line corresponds to the fit, while the red dashed lines are one standard deviation away, and the dotted black lines are two standard deviations away. (TIFF) [file pone.0036624.s002.tiff]

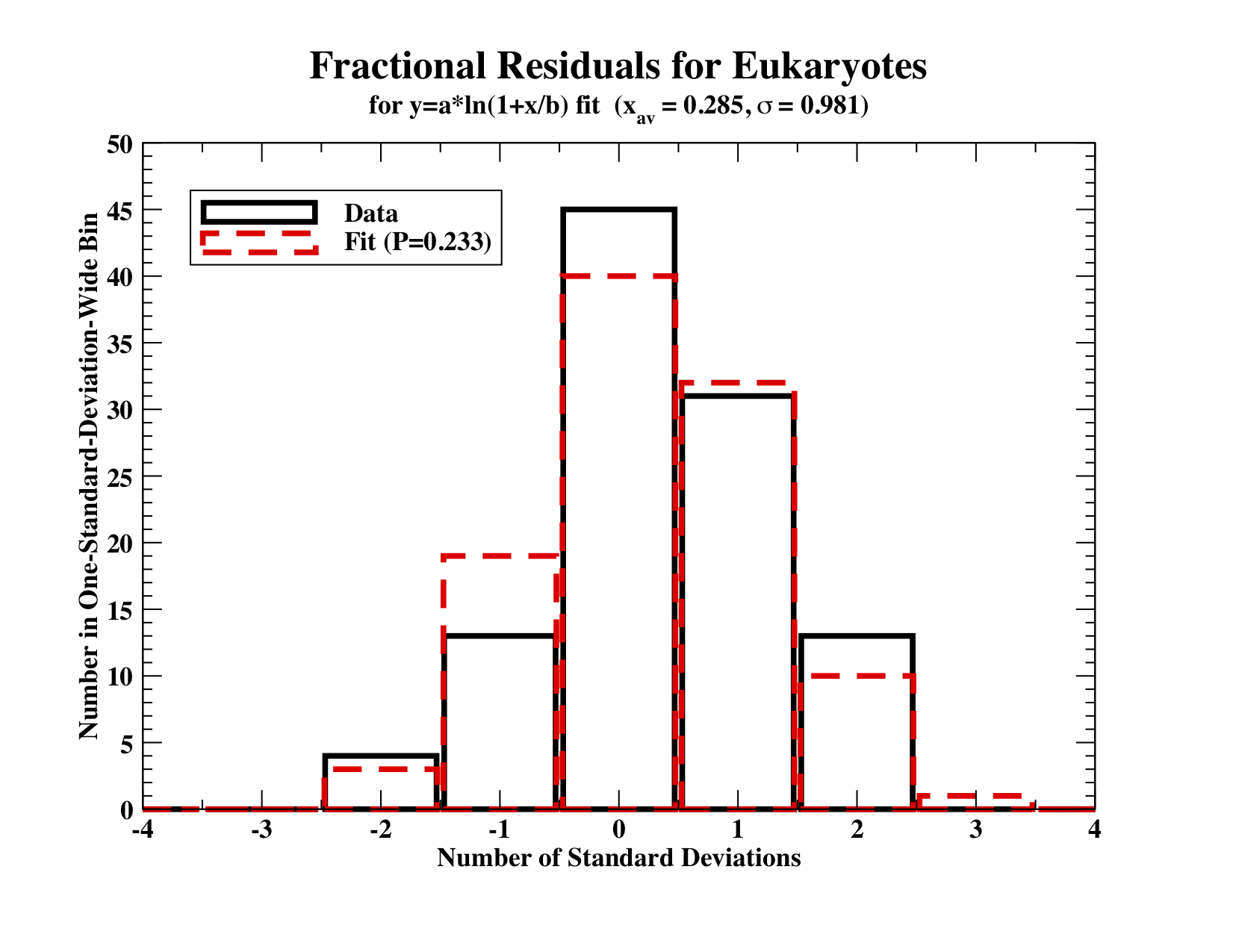

Supplement: Figure S3 — Binned Eukaryota Fractional Residuals. Eukaryota fractional residuals (in black) sorted into 7 one-standard-deviation-wide bins compared to an assumed Gaussian distribution with the same mean and variance (in red). (TIFF) [file pone.0036624.s003.tiff]

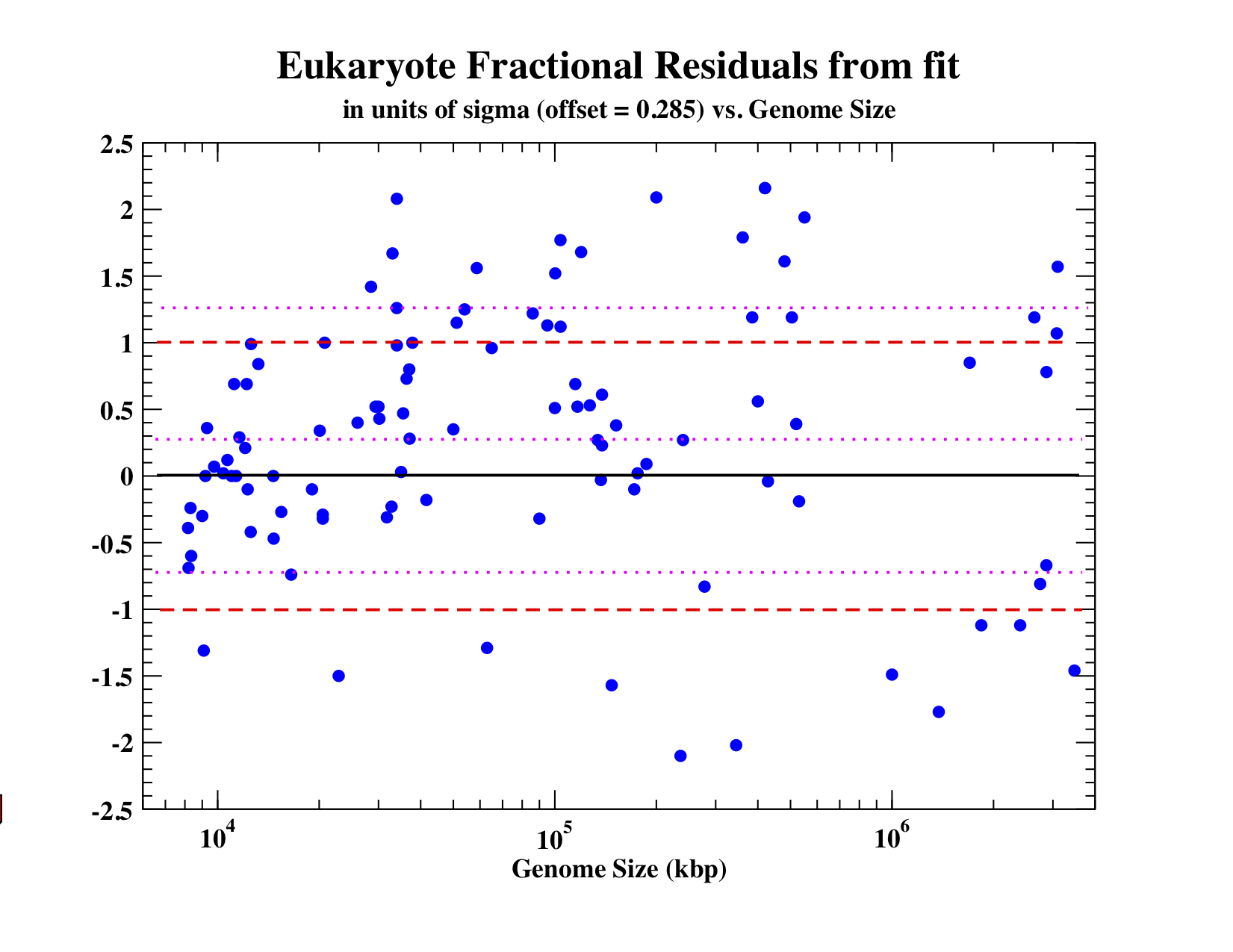

Supplement: Figure S4 — Eukaryota Fractional Residuals. Distribution of Eukaryota fractional residuals (in blue) as a function of genome size in kbp. The black line corresponds to the fit, while the red dashed lines are one standard deviation away. The dotted magenta line at 0.285 is the mean of the fractional residuals in units of (one) standard deviation, while the other two dotted magenta lines are one standard deviation away from the mean. (TIFF) [file pone.0036624.s004.tiff]
