# Supplementary material for: Genome Sizes and the Benford Distribution
Source: PLoS One. 2012 May 18;7(5):e36624. doi: 10.1371/journal.pone.0036624 (PMC3356352; doi:10.1371/journal.pone.0036624)
Supplement: Material S1 — Material on Fits, Fitting Procedures, and Statistics. Detailed notes and derivations. (PDF) [file pone.0036624.s005.pdf]

SUPPLEMENTARY MATERIAL

Our Fits and Fitting Procedures

CONTENTS

I. Introduction
II. Functionals
III. Minimization
IV. Formal Error Analysis
V. Practical Error Analysis
VI. Goodness of Fit and Tests of Significance
VII. Statistical Distributions Associated with Hypothesis Testing
VIII. Analysis of Fractional Residuals

I. Introduction

Because different areas of science have their own notations, their own tacit assumptions, and various methodological approaches, it was felt necessary to provide some background (as well as the details) about our fitting procedures. These procedures were implemented in a self-written, stand-alone Fortran program (**chi.f**) and not by a "canned" program. Our original area of physics (nuclear and particle physics) uses χ^2 minimization for almost all fits, because errors are well characterized. Least-squares techniques are used much less often, and the word *regression* seldom at all. The redundant nature of these notes is predicated upon that hierarchy, and will attempt to explain differences and consequences of various approaches. Generalities and background concerning fitting procedures (primarily derivations) are discussed in **red sections**. Specifics concerning how fitting and error analysis were performed for the results given in our paper and how we benchmarked our code against "canned" codes are primarily described in **Practical Error Analysis** (Section V), with amplification in subsequent **blue sections**. Assumptions about the uncertainties in our data are discussed in the paragraph above and the paragraph below Eqn. (34). Justification for these assumptions is given in Section V for Prokaryotes and in Section VIII for Eukaryotes.

II. Functionals

The (standard) ordinary least-squares functional is given by

$$LS\{a_M\} = \sum_{i=1}^{N} \left(y_i - F(x_i, \{a_M\})\right)^2 , \tag{1}$$

where y_i is the ith datum (one of N) corresponding to the variable point x_i and $F(x_i, \{a_M\})$ is the function that we wish to fit to the data and that depends on a set of M parameters denoted by $\{a_M\}$. Note that the LS functional in general has dimensions. We perform the fit by minimizing the LS functional with respect to each parameter a_α, which generates M simultaneous equations to be solved:

$$\frac{\partial\, LS\{a_M\}}{\partial a_\alpha} = 0\,. \qquad\qquad \alpha = 1, \cdots, M \tag{2}$$

This is an exceptionally common procedure and indeed is the set of equations used by the plotting package XMGRACE (that we employ) and the regression package in MATHEMATICA. The resulting fit parameters inserted in Eqn. (1) produce the sum of the squares of the residuals. This should be most sensitive to the largest values of y_i (which typically will have the largest residuals), particularly if the values of y_i have a large range. We note that this procedure is often called *regression analysis* in many areas of application, although that term is rarely used in nuclear and particle physics.

The fact that the LS functional has dimensions strongly suggests that an additional assumption is needed in order to perform error analysis. While most physical quantities and their errors have dimensions, the framework for extracting errors typically involves probability distributions, which are dimensionless. Indeed, Hoel in Chapter 8 of [1] states: "Least squares alone is capable of estimating the parameters only." A dimensionless functional such as χ^2 (below) is not limited in this sense. See also the discussion below Eqn. (31) and Ref. [2]. We will follow conventional physics usage and use the words *error* and *uncertainty* interchangeably, as we will the terms *Normal distribution* and *Gaussian distribution*.

Most physics applications minimize the **dimensionless** χ^2 functional (see Chapter 14 of Ref. [2] for a comprehensive discussion of relevant numerical techniques). This follows rigorously from maximizing the (probability) likelihood function $\mathcal{L}$ when the data are samples from independently distributed Gaussian random variables ($\mathcal{L} \propto \exp\left(-\chi^2/2\right)$), where

$$\chi^2\{a_M\} = \sum_{i=1}^{N} \left(\frac{y_i - F(x_i, \{a_M\})}{\sigma_i} \right)^2 , \tag{3}$$

$$\frac{\partial\, \chi^2\{a_M\}}{\partial a_\alpha} = 0\,, \qquad\qquad \alpha = 1, \cdots, M \tag{4}$$

and σ_i is the standard error for the point (x_i, y_i) that provides the additional scale needed to perform error analysis. This form is also intuitively satisfying because terms with large uncertainties should have correspondingly less weight in the fit. Note that the Central Limit Theorem (if applicable) will tend to force other (than Gaussian) independently and identically distributed random variables into the form given in Eqn. (3).

For purposes of completeness it's worth comparing this to extensions of the simplest least-squares procedure. If we introduce weights, w_i, to the individual terms in the sum in Eqn. (1), the generalized (or weighted) least-squares functional and its minimization condition become

$$GLS\{a_M\} = \sum_{i=1}^{N} w_i \left(y_i - F(x_i, \{a_M\})\right)^2 , \tag{5}$$

and

$$\frac{\partial\, GLS\{a_M\}}{\partial a_\alpha} = 0 \, , \qquad \alpha = 1, \cdots, M \tag{6}$$

which is obviously equivalent to the usual χ^2 functional with $w_i = 1/\sigma_i^2$. For purposes of minimization all three forms are equivalent if we first set $\sigma_i \equiv \sigma$ (where σ is a constant) and $w_i \equiv w$ (where w is a constant), since Eqns. (4) and (6) are not affected by an overall constant. We will frequently use the convenient, compact form in Eqn. (5) to construct proofs, although our final results (and our entire approach) will be predicated on the χ^2 form.

III. Minimization

We perform the minimization by first expanding $F(x_i, \{a_M\})$ in each parameter a_α. Three values of a_α are noted: (1) a_α denotes an arbitrary value; (2) $\bar{a}_\alpha$ denotes the value at the minimum of the fitting procedure; (3) $\mathbf{a}_\alpha$ denotes the precise value of a_α in an exactly known formula (e.g., c in Einstein's relationship: $E = mc^2$). We also presume for later purposes of error analysis that the point y_i is a sample from a random variable, and that $y_i = \mathbf{y}_i + \hat{\epsilon}_i$, where $\mathbf{y}_i$ is the average of y_i in the limit of infinite samples taken, and $\hat{\epsilon}_i$ is the random error associated with y_i and has variance, $\overline{\hat{\epsilon}_i \hat{\epsilon}_j} = \delta_{ij}\sigma_i^2$. For convenience we also assume that $\overline{\hat{\epsilon}_i} = 0$. Clearly we also have $\mathbf{y}_i = F(x_i, \{\mathbf{a}_M\})$ if the formula, parameters, and data are exact.

We first treat the minimization of arbitrary nonlinear functionals for generalized least squares, which subsumes the other two cases. This makes the formulae slightly less messy than the full χ^2 case without affecting its

generality. We have to begin the minimization procedure with some initial guess for $\{a_M\}$ and then iterate to a solution by first expanding F using $a_\alpha \to a_\alpha + \delta a_\alpha$, keeping powers of δa_α in GLS through quadratic terms, and then solving Eqns. (6):

$$\begin{aligned} GLS\{a_M\} \;\approx\; & \sum_{i=1}^{N} w_i\,(y_i - F(x_i,\{a_M\}))^2 \\ -\; & 2\sum_{i=1}^{N} w_i\,(y_i - F(x_i,\{a_M\}))\sum_{\alpha=1}^{M}\frac{\partial F(x_i,\{a_M\})}{\partial a_\alpha}\delta a_\alpha \\ +\; & \sum_{i=1}^{N} w_i \sum_{\alpha=1}^{M}\frac{\partial F(x_i,\{a_M\})}{\partial a_\alpha}\delta a_\alpha \sum_{\beta=1}^{M}\frac{\partial F(x_i,\{a_M\})}{\partial a_\beta}\delta a_\beta \\ -\; & \sum_{i=1}^{N} w_i\,(y_i - F(x_i,\{a_M\}))\sum_{\alpha=1}^{M}\sum_{\beta=1}^{M}\frac{\partial^2 F(x_i,\{a_M\})}{\partial a_\alpha\,\partial a_\beta}\delta a_\alpha \delta a_\beta \end{aligned} \tag{7}$$

Ignored higher-order (than second) terms in $\{\delta a_M\}$ can affect the rate of convergence to a solution, but not the final result. Defining

$$d_i^\alpha = \frac{\partial F(x_i,\{a_M\})}{\partial a_\alpha} \tag{8}$$

and

$$e_i^{\alpha\beta} = \frac{\partial^2 F(x_i,\{a_M\})}{\partial a_\alpha\,\partial a_\beta} \tag{9}$$

leads to a simplified matrix form, where we now use the summation convention (any repeated Greek index implies a sum over that index):

$$GLS\{a_M\} \approx C - 2B_\alpha\,\delta a_\alpha + (A_{\alpha\beta} - D_{\alpha\beta})\,\delta a_\alpha\,\delta a_\beta \tag{10}$$

with

$$A_{\alpha\beta} = \sum_{i=1}^{N} w_i d_i^\alpha d_i^\beta\,, \tag{11}$$

$$B_\alpha = \sum_{i=1}^{N} w_i d_i^\alpha (y_i - F(x_i,\{a_M\}))\,, \tag{12}$$

$$C = \sum_{i=1}^{N} w_i\,(y_i - F(x_i,\{a_M\}))^2\,, \tag{13}$$

$$D_{\alpha\beta} = \sum_{i=1}^{N} w_i \, e_i^{\alpha\beta} (y_i - F(x_i, \{a_M\})) \,. \tag{14}$$

Applying Eqn. (6) leads to

$$(A_{\alpha\beta} - D_{\alpha\beta}) \, \delta a_\beta = B_\alpha \tag{15}$$

and for the moment dropping $D_{\alpha\beta}$ (which can not occur for linear fitting parameters) we find

$$\delta a_\alpha = A^{-1}_{\alpha\beta} \, B_\beta \,. \tag{16}$$

Adding δa_α to the current a_α to form a new a_α (for all α) and repeating the process in Eqns. (7)-(16) will recurse to a solution set $\{\bar{a}_M\}$ with any desired accuracy. The matrix $D_{\alpha\beta}$ vanishes for Linear Least Squares (LLS), and for small errors will be very small near a solution, unlike $A_{\alpha\beta}$. The former matrix can change the rate of convergence to a solution (as will the higher-order terms in δa_α), but will not affect the final solution. At the true minimum in every parameter a_α, all terms linear in δa_α must vanish, requiring

$$B_\alpha \{\bar{a}_M\} \equiv 0 \,, \tag{17}$$

which **defines** the minimum, and therefore

$$GLS\{\bar{a}_M + \delta a_M\} \approx C\{\bar{a}_M\} + (A\{\bar{a}_M\}_{\alpha\beta} - D\{\bar{a}_M\}_{\alpha\beta}) \, \delta a_\alpha \, \delta a_\beta \,. \tag{18}$$

This is the standard ellipsoidal χ^2 surface for small changes in fitted parameters about the minimum, which also determines the parameter errors. The discussion below Eqn. (25) shows that we can neglect the D term, which is standardly done.

We finally note that if F is a **linear** function of the a_α, then $F_{\rm lin}(x_i, \{a_M\}) \equiv \sum_\alpha a_\alpha \, d_i^\alpha$ and Eqns. (12) and (17) imply (by forming $\sum_\alpha a_\alpha B_\alpha$)

$$\sum_{i=1}^{N} w_i \, F_{\rm lin}(x_i, \{\bar{a}_M\}) \, (y_i - F_{\rm lin}(x_i, \{\bar{a}_M\})) = 0 \,, \tag{19}$$

or $\overline{yF}_{\rm lin} = \overline{F}^2_{\rm lin}$, which is essential for proving certain theorems involving regression. Another useful version of Eqn. (17) follows if one of the linear parameters multiplies a **constant** (i.e., d_i^β is independent of i for some β):

$$\sum_{i=1}^{N} w_i \, (y_i - F_{\rm lin}(x_i, \{\bar{a}_M\})) = 0 \,, \tag{20}$$

or $\bar{y} = \bar{F}_{\rm lin}$. Note that if w_i is independent of i this leads to the usual LLS statement that the sum of the residuals vanishes. In our case this condition is definitely not true (see Eqn. (34)), and since $w_i = 1/\sigma_i^2$, the sum in Eqn. (20) is also not the sum of fractional residuals, where each fractional residual is defined by

$$r_i = \frac{y_i - F(x_i, \{\bar{a}_M\})}{\sigma_i} \,. \tag{21}$$

IV. Formal Error Analysis

Fitted values of χ^2 are expected to increase by 1 for every datum added, and to decrease by 1 for every additional fitting parameter added:

$$\overline{\chi^2} = N - M \equiv N_{\rm dof} \,, \tag{22}$$

where $N_{\rm dof}$ defines the number of degrees of freedom. This intuitively makes sense since each data point y_i on the average should be about σ_i away from the infinite sample limit (see Eqn. (21)), and each fitted parameter "uses" one linear combination of data points. Moreover, K data points (regardless of random fluctuations) can be fitted exactly by a Kth-order polynomial, leading to the obvious result that a vanishing $\overline{\chi^2}$ requires vanishing $N_{\rm dof}$ (i.e., $N = M = K$).

To prove Eqn. (22) we now write y_i as a random variable ($y_i = \mathbf{y}_i + \hat{\epsilon}_i$) in Eqns. (10)-(21). We expand $\{a_M\}$ about the exact values associated with no random fluctuations: $\{\mathbf{a}_M\}$, with the requirement that $\mathbf{y}_i = F(x_i, \{\mathbf{a}_M\})$ because we are assuming that our fitting formula is exact. This means that all variables in our fitting functional are random variables (indicated by the *hats*), as is the *GLS* functional

$$\begin{aligned} GLS &\approx \sum_{i=1}^{N} w_i \left(-d_i^\alpha \delta\hat{a}_\alpha - \tfrac{1}{2} e_i^{\alpha\beta}\, \delta\hat{a}_\alpha\, \delta\hat{a}_\beta + \hat{\epsilon}_i \right)^2 \\ &\approx \sum_{i=1}^{N} w_i \hat{\epsilon}_i^2 - 2\, \hat{E}_\alpha\, \delta\hat{a}_\alpha + (A_{\alpha\beta} - \hat{F}_{\alpha\beta})\, \delta\hat{a}_\alpha\, \delta\hat{a}_\beta \,, \end{aligned} \tag{23}$$

where we have defined

$$\hat{E}_\alpha = \sum_{i=1}^{N} w_i\, d_i^\alpha\, \hat{\epsilon}_i \,, \tag{24}$$

and

$$\hat{F}_{\alpha\beta} = \sum_{i=1}^{N} w_i(\hat{\epsilon}_i - d_i^{\gamma}\, \delta\hat{a}_{\gamma})e_i^{\alpha\beta}. \tag{25}$$

At this point we can discard the $\hat{F}_{\alpha\beta}$ term in Eqn. (23), since the second part generates an explicitly third-order term in $\delta\hat{a}_{\alpha}$, and the first term together with the $\delta\hat{a}_{\alpha}\, \delta\hat{a}_{\beta}$ terms in Eqn. (23) are third order in the random error $\hat{\epsilon}_i$, and will produce a (higher-order) term proportional to the *skewness* of the error. Thus if we explicitly ignore all third-order terms in the errors and minimize GLS with respect to $\delta\hat{a}_{\alpha}$, we obtain

$$\delta\hat{a}_{\alpha} = A^{-1}_{\alpha\beta}\, \hat{E}_{\beta}\,, \tag{26}$$

and substituting Eqn. (26) into Eqn. (23) produces the solution

$$GLS\{\mathbf{a}_M + \delta\hat{a}_M\} = \sum_{i=1}^{N} w_i\hat{\epsilon}_i^2 - \hat{E}_{\alpha}\, A^{-1}_{\alpha\beta}\, \hat{E}_{\beta}\,. \tag{27}$$

Taking the expectation value of Eqn. (27) leads to the result in Eqn. (22) if we require only that $w_i = 1/\sigma_i^2$ (i.e., as in the χ^2 functional)

$$\overline{GLS\{\mathbf{a}_M + \delta\hat{a}_M\}} = N - M\,, \tag{28}$$

with the first and second terms in Eqn. (27) producing the corresponding terms on the right-hand side of Eqn. (28). Note that $\overline{\delta\hat{a}_{\alpha}}$ is non-vanishing if $\overline{\hat{\epsilon}_i} \neq 0$. A similar analysis using Eqn. (26) leads to the very useful relationship at the minimum

$$\overline{\delta\hat{a}_{\alpha}\ \delta\hat{a}_{\beta}} = A^{-1}_{\alpha\beta}\,, \tag{29}$$

where $A^{-1}_{\alpha\beta}$ is the very important **error matrix**. The diagonal elements are the variances of the parameters

$$\mathrm{var}(\hat{a}_{\alpha}) = \overline{\delta\hat{a}_{\alpha}\ \delta\hat{a}_{\alpha}} = A^{-1}_{\alpha\alpha}\,. \tag{30}$$

If we assume that $A_{\alpha\beta}$ is diagonal (and thus also $A^{-1}_{\alpha\beta}$), Eqns. (30) and (18) imply that χ^2 changes by 1 when a_{α} changes by ± 1 standard deviation (viz., 1 sd $= \sqrt{\overline{\delta\hat{a}_{\alpha}^2}} = \sqrt{A^{-1}_{\alpha\alpha}}$).

V. Practical Error Analysis

For the ordinary least-squares procedure one associates a constant error ($\sigma_i = \sigma$) with each data point y_i. This formal mapping of least squares into a χ^2 form does not affect the solution of the minimization equations, but Eqn. (22) ($\overline{\chi^2} = N - M \equiv N_{\text{dof}}$) then tells us what σ must be in order to perform an error analysis:

$$\sigma^2 = \frac{\sum_{i=1}^{N} (y_i - F(x_i, \{\bar{a}_M\}))^2}{N_{\text{dof}}} \,, \tag{31}$$

where again $\bar{a}_M$ is the fitted value of a_M. Note that Eqn. (31) is also the maximum likelihood estimate for σ, assuming independently and identically distributed (denoted **i.i.d.**) Gaussian random variables (see Section 8.5 of Hoel [1]). This is in fact the conventional form of the variance, and it is difficult to imagine any expression more reasonable for a variance in this case. However, Eqn. (31) is sometimes imposed in the ordinary least-squares procedure by conflating without proof the (statistical) variance with a measure of the spread in the data. In Ref. [2] (p. 503) it is stated: "In some cases the uncertainties associated with a set of measurements are not known in advance, and considerations related to χ^2 fitting are used to derive a value for σ. If we assume that all measurements have the same standard deviation, $\sigma_i = \sigma$, and that the model does fit well, then we can proceed by first assigning an arbitrary constant σ to all points, next fitting for the model parameters by minimizing χ^2, and finally recomputing [Eqn. (31)]. Obviously this approach prohibits an independent assessment of goodness-of-fit, a fact occasionally missed by its adherents. When, however, the measurement error is not known, this approach at least allows *some* kind of error bar to be assigned to the points."

In the more general χ^2 case we have previously defined the fractional residual (which is obviously dimensionless) for each point

$$r_i \equiv \frac{y_i - F(x_i, \{\bar{a}_M\})}{\sigma_i} \,, \tag{32}$$

in terms of which Eqn. (22) can be written as

$$\chi^2_{\text{min}} = \sum_{i=1}^{N} r_i^2 = N_{\text{dof}} \,. \tag{33}$$

This means that the variance of the fitted (fractional) residuals is minimized, and consequently the fractional residuals can (in general) have a non-vanishing mean.

Error analysis can be completed by using Eqn. (30). This generates δa_M, and the fit parameter has the final value and uncertainty: $\bar{a}_M \pm \delta a_M$. We have verified for several test cases (corresponding to constant errors) that the XMGRACE plotting package (that we use) and the MATHEMATICA regression package produce parameters in agreement with ours and errors in agreement with Eqns. (22) and (31), and thus **assumes** this prescription for generating parameter uncertainties associated with Eqn. (1). Note again that this is a prescription, because it is used regardless of how the data are distributed.

If uniform errors are inappropriate, one can try uniform fractional errors. This is what we have assumed for the uncertainty in the number of ORFs in each genome. It is also the more usual case in nuclear and particle physics, where counting statistics corresponds to independently distributed random variables, and experiments are designed to make individual measurements comparable in fractional error wherever possible (e.g., in the measurement of a form factor or cross section) in order to make each datum of comparable importance. Uniform fractional errors correspond to

$$\sigma_i = \lambda\, y_i \,, \tag{34}$$

and one uses Eqns. (3) and (4) to determine the fitting parameters and Eqn. (22) to determine λ

$$\lambda^2 = \left[\frac{1}{N_{\rm dof}}\right] \sum_{i=1}^{N} \left(\frac{y_i - F(x_i, \{\bar{a}_M\})}{y_i}\right)^2 . \tag{35}$$

If we assume that the data are independently distributed Gaussian random variables, the likelihood function is given by

$$\mathcal{L} = \prod_{i=1}^{N} \frac{1}{\sqrt{2\pi}\,\lambda y_i} \exp\left(-\frac{1}{2}\left(\frac{y_i - F(x_i, \{a_M\})}{\lambda\, y_i}\right)^2\right) , \tag{36}$$

where we have used Eqn. (34) for σ_i. The logarithm of this function is

$$\ln \mathcal{L} = -N \ln(\sqrt{2\pi}\, y_i) - N \ln\lambda - \frac{1}{2}\sum_{i=1}^{N}\left(\frac{y_i - F(x_i, \{a_M\})}{\lambda\, y_i}\right)^2 , \tag{37}$$

and setting its derivative with respect to λ to zero yields Eqn. (35) with $N_{\rm dof}$ replaced by N. Equation (35) is the better (unbiased) estimate. Thus the **maximum likelihood estimate** for λ and forcing the minimum χ^2 to equal $N_{\rm dof}$ are equivalent; this is the analogue of Eqn. (31) for ordinary least squares. Note that both the minimization of χ^2 with respect to the parameters and the determination of λ were obtained using the **maximum likelihood method**.

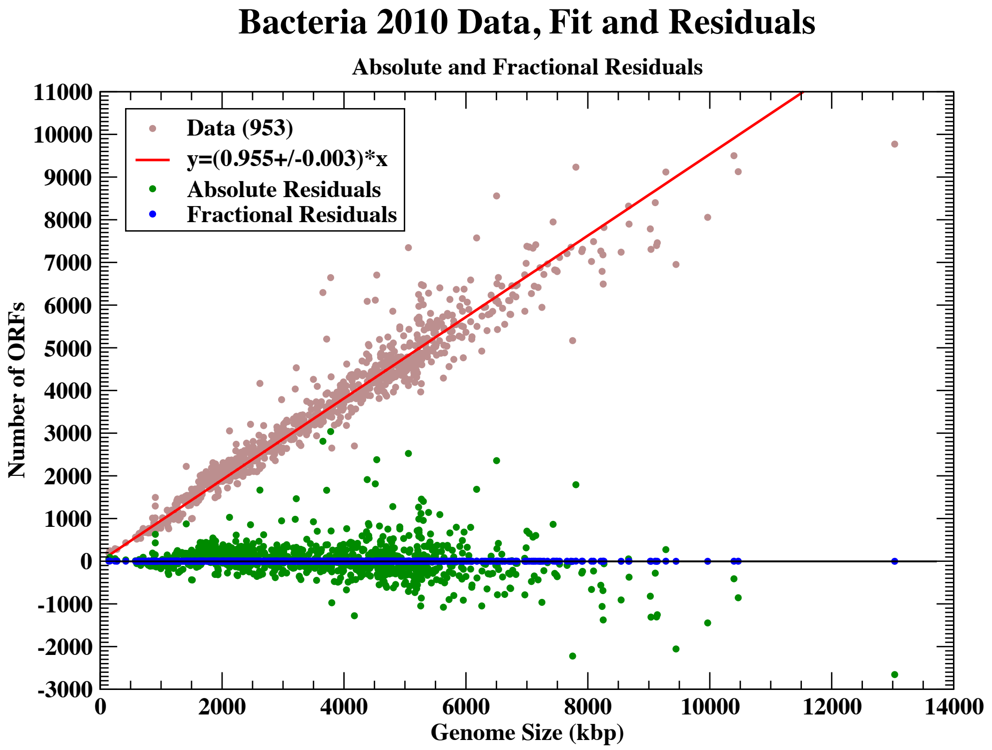

Figure 1: **Bacteria Data, Fits and Residuals.** Distribution of Bacteria data about the fit line (in red) and absolute residuals relative to that line (in green). Fractional residuals (using Eqns. (32) and (34)) are shown in blue.

An examination of the residuals set for one of our cases in Fig. (1) shows that the fractional residuals corresponding to constant errors ($\sigma_i = \sigma$) are tiny for small x_i and very large for large x_i. A constant error does not there-

fore lead to residuals that have a reasonably constant variance (the technical term is homoscedasticity), nor does it reflect the actual uncertainty in the number of ORFs as a function of genome size. Whatever uncertainties exist in the determination of the number of ORFs in any genome, they are almost certain to be determined by the size of that genome (we have assumed a linear dependence), and not be a constant independent of genome size. The latter would completely suppress the influence of small genomes and exaggerate the importance of large ones. This is especially true for the Eukaryota case, where genome sizes extend over more than four decades (justification of uniform fractional uncertainties for Eukaryotes is treated in Section VIII). Assuming uniform fractional errors also makes the influence of each datum more "democratic."

The raw residuals corresponding to fits that assume a common fractional error have a similar behavior to the case above, while the fractional residuals that include the factor of σ_i given by Eqn. (34) do indeed have the *a priori* reasonable-looking (statistical) distribution shown in Fig. (2). More importantly they have a sensibly constant variance as a function of genome size. There are obvious and problematic outliers (defined as points more than 3 standard deviations from the fit line, with a total probability of $\approx 1/4\%$ for a Gaussian distribution). Although their number is small (approximately 2% of the total number of data points, and the reason for their existence unknown), there are still far too many to be generated by a Gaussian distribution. Clumping of points in the distribution is likely due to selection bias in the choice of genomes to be sequenced. Note that we also assume that errors associated with the x variable are negligible compared to those associated with y (viz., σ_i).

We will nevertheless assume that the distribution of fractional residuals corresponds to a probability distribution, and by default this will be taken to be a Gaussian distribution. We will test this assumption below (see Fig. (3)) by projecting all data for each domain of life (regardless of genome size) onto the y-axis to generate domain-wide residuals sets. We will find that the Eukaryota data set tests better against a Gaussian distribution than does the Bacteria set (because of the outliers in the latter).

VI. Goodness of Fit and Tests of Significance

The conventional test of "goodness of fit" in physics applications is the value of χ^2 per degree of freedom that results after the fit is performed

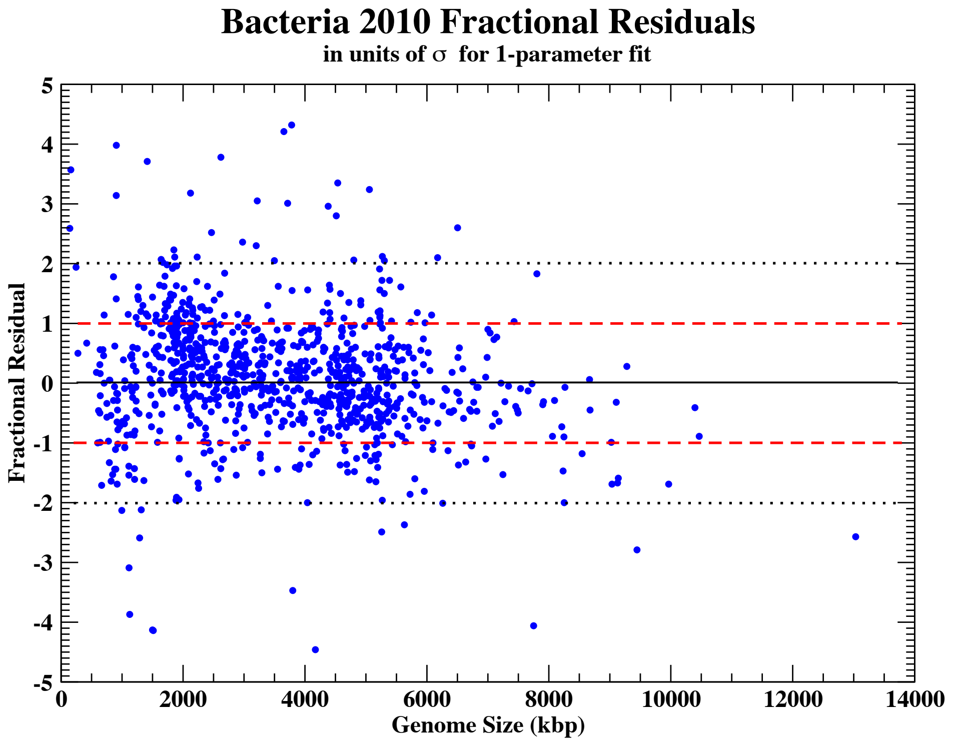

Figure 2: **Fractional Bacteria Residuals.** Distribution of fractional Bacteria residuals (in blue) is shown relative to the fit line. The dashed red line corresponds to $\pm$ 1 standard deviation, while the dotted black line depicts $\pm$ 2 standard deviations.

(compared to 1). Because we don't have well-characterized errors in this problem, we used the maximum likelihood estimate for any unknown error parameters such as σ (for constant errors) or λ in our actual problem (which is exactly equivalent to making the effective χ^2 per degree of freedom equal to 1). This eliminates any direct goodness of fit test from χ^2. One can, however, choose a **particular** fit and value of λ to "complete" the data set with fixed errors, and judge subsequent fits by the changes in χ^2 (greater χ^2 means a worse fit, and smaller χ^2 means a better fit). Equivalently one can judge subsequent fits according to whether λ goes up (a worse fit) or down (a better fit).

One measure of "goodness of fit" that is commonly reported for individual fitted parameters in a regression is the t-statistic (indeed, the MATHEMATICA regression package automatically provides this). This is more accurately described as a test of statistical significance. By dividing the fitted parameter value ($\bar{a}$) by its standard deviation, δa, (i.e., $t = \bar{a}/\delta a$), this statistic is nothing more than the number of standard deviations that the parameter varies from zero. One then uses the one-sided Student t-distribution (see Section VII) to determine a P-value for that t-statistic and N_{dof} degrees of freedom. For fits of our quality (t is a number much, much larger than 1), this value will be negligibly small, implying that the probability is negligibly small that random fluctuations in the data sample can drive the parameter to its fitted value. For practical purposes a t-parameter value of 2 (or higher) means that there is only a 5% chance (or less) that fluctuations can achieve that value. We have verified that the MATHEMATICA regression package reproduces the t-statistics and P-values for individual parameters from our fitting code for the case of constant errors.

We can easily extend this to calculating the variation in the entire fitted function, which is determined by the error matrix: $A^{-1}_{\alpha\beta}$. Expanding the function $F(x_i, \{a_M\})$ (in the a_α) about the fitted values $\{\bar{a}_\alpha\}$ by the small random amounts $\delta\hat{a}_\alpha$ (driven by the fluctuations in the data) produces

$$\delta\hat{F}_i \equiv F(x_i, \{a_M\}) - F(x_i, \{\bar{a}_M\}) \cong \sum_\alpha d_i^\alpha \delta\hat{a}_\alpha \,, \tag{38}$$

using Eqn. (9). Computing the variance and using Eqn. (29), which defines the error matrix, leads to

$$\overline{(\delta\hat{F}_i)^2} = \sum_{\alpha,\beta} d_i^\alpha \, A^{-1}_{\alpha\beta} \, d_i^\beta \,. \tag{39}$$

We can therefore easily compute an analogue t-statistic for the fitted function at the $i\underline{th}$ data point by dividing the fitted function at that point by its standard deviation

$$t_i = F(x_i, \{\bar{a}_M\}) / \sqrt{\overline{(\delta \hat{F}_i)^2}}\,, \tag{40}$$

and finally average these values over all N data points

$$\bar{t} = \frac{\sum_i t_i}{N}\,. \tag{41}$$

Our t-statistic $\bar{t}$ can now be processed in the usual way. Our smallest $\bar{t}$ is larger than 15, which corresponds to a negligible P-value. We note that if the fit involves only a single linear fitting parameter a (i.e., $F(x_i, a) = a \cdot g(x_i)$, where the function g is arbitrary) our t-statistic reduces to $\bar{t} = \bar{a}/\delta a$, which is the usual single-parameter result.

A parameter that is commonly calculated in the context of least-squares fits is the "Coefficient of Determination", R^2, which is the square of the Pearson Product-Moment Correlation Coefficient, r. We will use the notation r^2 instead of R^2. There are three versions of r^2 that pertain to least-squares fits. If the parameter dependence is linear, they are identical (but not otherwise), as we will derive below. Note that "linear in parameters" does not necessarily have the same meaning as "straight-line fit".

We first define a simplified notation that makes presentation more transparent:

$$f_i \equiv F(x_i, \{\bar{a}_M\})\,. \tag{42}$$

The mean, $\bar{y}$, of the data, y_i, is defined by

$$\bar{y} = \frac{\sum_{i=1}^{N} \; w_i \, y_i}{\sum_{i=1}^{N} \; w_i}\,, \tag{43}$$

and

$$\bar{f} = \frac{\sum_{i=1}^{N} \; w_i \, f_i}{\sum_{i=1}^{N} \; w_i}\,. \tag{44}$$

We note that if we use the fractional errors of Eqn. (34) for σ_i, then $\bar{y} = (\sum_{i=1}^{N} \; 1/y_i)/(\sum_{i=1}^{N} \; 1/y_i^2)$ and is weighted toward smaller values of y_i.

We first form

$$SS_{\rm err} + SS_{\rm reg} \equiv \sum_{i=1}^{N} \; w_i \, (y_i - f_i)^2 + \sum_{i=1}^{N} \; w_i \, (f_i - \bar{y})^2 = SS_{\rm tot}\,, \tag{45}$$

and use Eqns. (19) and (20) (which imply $\bar{f} = \bar{y}$ and $\overline{fy} = \overline{f^2}$) to show that $SS_{\rm tot}$ equals $\sum_{i=1}^{N} \; w_i\,(y_i - \bar{y})^2$. We have used a fairly common notation for the first and second terms in the sum above (viz., the Sum of Squares of Residuals (or Errors: $SS_{\rm err}$) and Regression Sum of Squares ($SS_{\rm reg}$), respectively). These two terms sum to $SS_{\rm tot}$, which is the Total Sum of Squares. It is also common to call the Regression Sum of Squares the "explained variance", while the Sum of Squares of Residuals is denoted the "unexplained variance." This nomenclature is quite standard; see Ref. [3] for a lucid explanation. Rearranging Eqn. (45) leads to

$$1 = \frac{\sum_{i=1}^{N} \; w_i\,(y_i - f_i)^2}{\sum_{i=1}^{N} \; w_i\,(y_i - \bar{y})^2} + \frac{\sum_{i=1}^{N} \; w_i\,(f_i - \bar{y})^2}{\sum_{i=1}^{N} \; w_i\,(y_i - \bar{y})^2}\,, \tag{46}$$

and we define the last quantity in Eqn. (46) (viz., $SS_{\rm reg}/SS_{\rm tot}$, which is the fraction of the total variance that is "explained") to be

$$r^2 = \frac{\sum_{i=1}^{N} \; w_i\,(f_i - \bar{y})^2}{\sum_{i=1}^{N} \; w_i\,(y_i - \bar{y})^2}\,. \tag{47}$$

Equation (46) then generates an alternative expression for r^2 given by

$$r^2 = 1 - \frac{\sum_{i=1}^{N} \; w_i\,(y_i - f_i)^2}{\sum_{i=1}^{N} \; w_i\;(y_i - \bar{y})^2}\,, \tag{48}$$

expressed in terms of the residuals of the fit and the variance of y. Note that $r^2 = 1$ follows if each of the y_i is arbitrarily close to each of the $f_i \equiv F(x_i, \{\bar{a}_M\})$.

An alternative form for r (and the preferred one) is given by Pearson's product moment correlation coefficient

$$r = \frac{\sum_{i=1}^{N} \; w_i\,(y_i - \bar{y})(f_i - \bar{y})}{\sqrt{\sum_{i=1}^{N} \; w_i\,(y_i - \bar{y})^2 \sum_{i=1}^{N} \; w_i\,(f_i - \bar{y})^2}}\,. \tag{49}$$

If one adds and subtracts f_i in the first parenthesis bracket in the numerator, and then uses Eqns. (19) and (20), one obtains Eqn. (47). All three versions are cryptically listed on the website *Mathworld*, under the label "Correlation Index." Clearly r approaches 1 if each y_i is arbitrarily close to each of the $f_i \equiv F(x_i, \{\bar{a}_M\})$. Numerical tests show that Eqn. (49) is the most stable variant of r, followed by Eqn. (48). If there is no constant term in the fit

(violating the conditions for equality), then Eqn. (47) is slightly unstable, as well. This can affect the stability of the F-statistic that we calculate below. We further note that if we perform a straight-line fit (viz., $y = a + b \cdot x$) the two factors of $(f_i - \bar{y})$ in Eqn. (49) can be replaced by $(x_i - \bar{x})$, which is the usual form of Pearson's covariance for two random variables; the constants a and b cancel out (ONLY) in this case.

The equality of the three forms of r (i.e., Eqns. (47), (48), and (49)) holds only if F is a **linear** function of the a_α, and if one of those parameters determines a **constant** term in F. Otherwise the three forms can have different values. Note that the various quantities in Eqns. (47), (48), and (49) are independent of the overall scale in w_i, such as λ in Eqn. (34). We also note that r will be sensitive to outliers in the data, given the quadratic nature of each term.

Our final entry in this section is more a test of (statistical) significance than of goodness of fit. In principle the statistical fluctuations inherent in sampling could account for the data that we fit even if there were no actual relationship between the data and the model. One typically adopts the null hypothesis that all of the fitted coefficients are zero, and tests this by constructing the appropriate test-statistic and the corresponding probability that the null hypothesis is true. By convention probabilities less than 5% are assumed to reject the null hypothesis (i.e., the probability of statistical fluctuations producing the result is too small to be significant).

A very common procedure is to construct the ratio of the "explained" variance to the "unexplained" variance (viz., $SS_{\text{reg}}/SS_{\text{err}} = \frac{r^2}{1-r^2}$) as the basis for a test-statistic, and use this to test the significance of r (see the text above Eqn. (47) and recall that $r^2 \equiv R^2$, with the latter being the more usual notation). As shown in Chapter 11 of Hoel [1], two scaled χ^2 distributions (e.g., variances) u (with ν_1 degrees of freedom) and v (with ν_2 degrees of freedom) can be tested by forming the F-statistic as input to the F-distribution (named in honor of R. A. Fisher - see next section), with argument F and indexes ν_1 and ν_2

$$F \equiv \frac{\frac{u}{\nu_1}}{\frac{v}{\nu_2}} = \left[\frac{\nu_2}{\nu_1}\right]\left[\frac{u}{v}\right] = \left[\frac{N_{\text{dof}}}{M-1}\right]\frac{r^2}{1-r^2}\ . \tag{50}$$

We have used the appropriate values: $\nu_1 = M - 1$ and $\nu_2 = N_{\text{dof}}$. Note that ν_1 and ν_2 are often called the **numerator** and **denominator** degrees of freedom, for obvious reasons. For all of our cases the number of fitting

parameters, M, is small (typically, a few), the number of degrees of freedom, $N_{\rm dof}$, is large (> 50), and and the correlation coefficient, r, is close to 1. For these cases an F-statistic $\gtrsim 4$ signifies a P-value < 0.05. Our **smallest** F-statistic is 450, which generates a minuscule P-value. This is no surprise, since a superficial inspection of the graph with the data and the fits demonstrates that fluctuations could not possibly account for the results.

We have verified (for constant errors and both linear and non-linear fits) that our results for r are identical to those of the XMGRACE regression package (called the "correlation coefficient" by XMGRACE). For the linear fits our results for F also agree (XMGRACE does not compute this statistic for non-linear fits).

VII. Statistical Distributions Associated with Hypothesis Testing

One of the central problems of statistics is to determine the probability distribution of a function of a random variable. In common usage one starts with a Normal distribution and this leads to a variety of other distributions (depending on the application); this often involves the distribution of ratios of random variables. Reference [1] proves a number of theorems that summarize most situations that commonly arise. A useful notation is $N(\mu, \sigma^2)$ for a random variable distributed Normally with mean μ and variance σ^2. Four of these theorems state:

- If x is $N(0,1)$, then the sum of squares of N random samples of x has a χ^2 **distribution** with N degrees of freedom.
- If x is $N(\mu, \sigma^2)$ and s^2 is the sample variance based on N samples, then $N\,s^2/\sigma^2$ has a χ^2 **distribution** with $N-1$ degrees of freedom.
- If random variables u (u is $N(0,1)$) and v (v^2 has a χ^2 distribution with ν degrees of freedom) are independently distributed, then $t = u/[v/\sqrt{\nu}] = u\sqrt{\nu}/v$ has a **Student's t-distribution** with ν degrees of freedom.
- If u and v possess independent χ^2 distributions with ν_1 and ν_2 degrees of freedom, respectively, then $F = \frac{u/\nu_1}{v/\nu_2}$ has an **F-distribution** with ν_1 and ν_2 degrees of freedom.

The three derived distributions will be dealt with in succession. Note that *variances* have a χ^2 distribution and that *means* have a Normal distribution. Although we don't require it, the Cauchy distribution arises from the ratio of two $N(0,1)$ distributions.

The χ^2 **probability distribution function** for ν degrees of freedom has the form (see Ref. [1])

$$p(\chi^2, \nu) = \frac{(\chi^2)^{\frac{\nu}{2}-1} e^{-\frac{\chi^2}{2}}}{2^{\frac{\nu}{2}} \Gamma(\frac{\nu}{2})}\,, \tag{51}$$

and its cumulative distribution function is given by

$$P(\nu, \chi^2) = \frac{\gamma(\nu/2, \chi^2/2)}{\Gamma(\nu/2)}\,, \tag{52}$$

while the complement of the cumulative distribution function is given by

$$Q(\nu, \chi^2) = 1 - P(\nu, \chi^2)\,. \tag{53}$$

Student's t-distribution function for ν degrees of freedom is defined by the probability distribution function (see Ref. [1] for a derivation)

$$f(t) = \frac{1}{\sqrt{\nu}\, B\left(\frac{1}{2}, \frac{\nu}{2}\right)} \left(1 + \frac{t^2}{\nu}\right)^{-\frac{\nu+1}{2}}\,. \tag{54}$$

Its relevant (two-sided) cumulative distribution function is given by

$$A(t|\nu) = \frac{1}{\sqrt{\nu}\, B\left(\frac{1}{2}, \frac{\nu}{2}\right)} \int_{-t}^{t} \left((1 + \frac{x^2}{\nu}\right)^{-\frac{\nu+1}{2}} dx\,, \tag{55}$$

which is symmetric in t, normalized to $A(\infty|\nu) = 1$, and is not the usual cumulative distribution function. The latter integrates from $-\infty$ to t, rather than from $-t$ to t. Note that the symmetry in t means that the area under the curve f from t to ∞ is identical to that from $-\infty$ to $-t$. Therefore the complement of (the two-sided) A defined by

$$Q(t|\nu) = 1 - A(t|\nu) \tag{56}$$

is exactly twice the complement of the (usual or one-sided) cumulative distribution function. These two forms are also referred to as "two-tailed" or "one-tailed." Testing the statistical significance of results from fits typically requires the **one-sided** version, or $\frac{1}{2}Q(t|\nu)$. We note that after a change of

variables in Eqn. (55) to $1+\frac{x^2}{\nu}=1/z$ both $A(t|\nu)$ and $Q(t|\nu)$ can be written in terms of the usual incomplete (ratio) beta function defined by

$$I_x\left(\alpha,\beta\right)=\frac{1}{B(\alpha,\beta)}\int_0^x dz\, z^{\alpha-1}(1-z)^{\beta-1}\,, \tag{57}$$

with

$$Q(t|\nu)=I_{\frac{\nu}{\nu+t^2}}\left(\frac{\nu}{2},\frac{1}{2}\right)\,. \tag{58}$$

This quantity then determines the success or failure of any relevant hypothesis test, since $Q(t|\nu)$ (or $\frac{1}{2}Q(t|\nu)$) is the P-value or probability that fluctuations can lead to the values being tested. Subroutines for $I_x(\alpha,\beta)$ are readily available in Ref. [2]. Note that the integral for $I_x\left(\alpha,1\right)$ is trivial and we find $I_x\left(\alpha,1\right)=x^{\alpha}$.

The other distribution mentioned in the previous section was the **F probability distribution function** (sometimes called the variance-ratio distribution), which is defined over $[0,\infty]$ (see Ref. [1] for a derivation)

$$f(F)=\frac{1}{B\left(\frac{\nu_1}{2},\frac{\nu_2}{2}\right)\,F}\;\left(\frac{\nu_1}{\nu_2}F\right)^{\frac{\nu_1}{2}}\left(1+\frac{\nu_1}{\nu_2}F\right)^{-\frac{\nu_1+\nu_2}{2}}\,. \tag{59}$$

Its cumulative distribution function is therefore given by

$$C(F|\nu_1,\nu_2)=\frac{1}{B\left(\frac{\nu_1}{2},\frac{\nu_2}{2}\right)}\;\int_0^F\frac{dx}{x}\;\left(\frac{\nu_1}{\nu_2}x\right)^{\frac{\nu_1}{2}}\left(1+\frac{\nu_1}{\nu_2}x\right)^{-\frac{\nu_1+\nu_2}{2}}\,. \tag{60}$$

After changing variables to $\frac{\nu_1}{\nu_2}x=\frac{1}{z}-1$ we find

$$\begin{aligned}C(F|\nu_1,\nu_2)&=1-\frac{1}{B\left(\frac{\nu_1}{2},\frac{\nu_2}{2}\right)}\;\int_0^{\frac{\nu_2}{\nu_2+\nu_1F}}dz\;z^{\frac{\nu_2}{2}-1}\left(1-z\right)^{\frac{\nu_1}{2}-1}\\&=1-I_{\frac{\nu_2}{\nu_2+\nu_1F}}(\frac{\nu_2}{2},\frac{\nu_1}{2})\,.\end{aligned} \tag{61}$$

The complement of the cumulative distribution function is therefore

$$Q(F|\nu_1,\nu_2)=1-C(F|\nu_1,\nu_2)=I_{\frac{\nu_2}{\nu_2+\nu_1F}}(\frac{\nu_2}{2},\frac{\nu_1}{2})\,. \tag{62}$$

Note that $I_0(a,b)=0$ and $I_1(a,b)=1$, which guarantees that $C(F|\nu_1,\nu_2)$ is properly normalized. The cumulative distribution function $Q(F|\nu_1,\nu_2)$ then

determines the success or failure of any hypothesis test, since $Q(F|\nu_1, \nu_2)$ is the P-value or probability that fluctuations can lead to the values being tested.

We can also ask the question: "What critical value of F (i.e., $F_{\rm crit}$) corresponds to a P-value of α for a given set of ν_1 and ν_2?" The answer is given by the solution to

$$Q(F|\nu_1, \nu_2) = I_{\frac{\nu_2}{\nu_2+\nu_1 F}}(\frac{\nu_2}{2}, \frac{\nu_1}{2}) = \alpha \ . \tag{63}$$

Defining the solution of the equation

$$I_x(a, b) = \alpha \tag{64}$$

to be $x_\alpha = I_\alpha^{-1}(a, b)$, we find the answer of the question (for appropriate values of a and b) to be

$$F_{\rm crit} = \frac{\nu_2}{\nu_1} \left(\frac{1}{x_\alpha} - 1 \right) \ . \tag{65}$$

Because x in Eqn (64) varies from 0 to 1 and $I_x(a, b)$ varies monotonically over that range, it is a simple matter to program $I_\alpha^{-1}(a, b)$. **We find that for our set of fits any value of F greater than 4 generates a P-value smaller than 0.05.** Our **smallest** F-statistic was 450.

We observe that if we use $\nu_1 = 1$, $\nu_2 = \nu$, and $F = t^2$ in $Q(F|\nu_1, \nu_2)$ for the F-distribution, we obtain $Q(t|\nu)$ for the (two-sided) t-distribution. Student's t-distribution is therefore a special case of the more general F-distribution.

Our final task is to develop a simple way to assess P-values from the F-statistic, assuming that we have good fits (e.g., $r \gtrsim 0.9$) and many data used for a fit ($N \gg 1$ and $N \gg M$). We note that using multiple copies (e.g., K copies) of a datum at each value of x_i is equivalent to multiplying w_i (see Eqn. (5)) by K and N by K. Equation (49) demonstrates that r is independent of K and thus roughly independent of the number of data. The F-statistic in Eqn. (50), however, is explicitly proportional to K (i.e., proportional to $N_{\rm dof} \approx N$). This elementary exercise illustrates the fundamental difference between r (a goodness-of-fit measure) and F (a statistical significance measure), although some practitioners use *goodness-of-fit* to refer to both.

Using Eqn. (50) we can rewrite Equation (62) in terms of a parameter $\bar{x}$

$$\bar{x} = \frac{\nu_2}{\nu_2 + \nu_1 F} = 1 - r^2 \ , \tag{66}$$

and therefore

$$Q(F|\nu_1,\nu_2) = I_{1-r^2}\left(\frac{N_{\rm dof}}{2}, \frac{M-1}{2}\right) \ . \tag{67}$$

Note that although $1-r^2$ is roughly independent of the number of data, $I_{1-r^2}\left(\frac{N_{\rm dof}}{2}, \frac{M-1}{2}\right)$ is highly dependent on that number. We are interested in Eqn. (67) in the limit of large $N_{\rm dof}$ for fixed $1-r^2$ and $M-1$. The leading term in such an expansion in inverse powers of $N_{\rm dof}$ was developed in Ref. [4]:

$$I_{1-r^2}\left(\frac{N_{\rm dof}}{2}, \frac{M-1}{2}\right) \sim \frac{\Gamma\left(\frac{N_{\rm dof}+M-1}{2}\right)}{\Gamma\left(\frac{N_{\rm dof}}{2}+1\right)\,\Gamma\left(\frac{M-1}{2}\right)}\,(1-r^2)^{\frac{N_{\rm dof}}{2}}\, r^{M-3} \ . \tag{68}$$

A good fit with r close to 1 will generate minuscule P-values if the number of data is large because of the factor: $(1-r^2)^{\frac{N_{\rm dof}}{2}}$, which is the exact result for $M = 3$ (see below Eqn. (58)). Note again the difference between a *goodness-of-fit* test and a test of *statistical significance*. We also note that the asymptotic form in Eqn. (68) would have been different if we hadn't allowed for the dependence of F on $N_{\rm dof}$ (see Eqn. (50)).

We can perform the same type of analysis for the t-statistic that measures the number of standard deviations that a parameter deviates from zero: $\bar{a}/\delta a$. From the third bullet (above) we see that the variable $\bar{a}/\sigma_{\bar{a}} = \bar{a}\sqrt{N}/\sigma$ satisfies the conditions for the variable u, where σ^2 and $\sigma^2_{\bar{a}}$ are the stochastic population variance and the population variance per sample. Moreover, from bullet two we conclude that $v^2 = Ns^2/\sigma^2$ satisfies the conditions for v^2 with $\nu = N-1$ degrees of freedom, where $s^2 = N\,\delta a^2$ is the **sample** estimate of σ^2. This generates the appropriate t-statistic

$$t = \frac{\bar{a}\sqrt{N-1}}{s} \ . \tag{69}$$

This somewhat complicated process is necessary in order to obtain $\sqrt{N-1}$ rather than $\sqrt{N}$ in Eqn. (69). If this distinction is unimportant, then $\bar{a}/\delta a$ is immediately seen to be equivalent to Eqn. (69). Equation (30) demonstrates that if we insert K copies of each datum into our analysis then δa scales by $1/\sqrt{K}$, which is the usual $1/\sqrt{N}$ factor that arises in data analysis for N samples. Note that s does not scale with the number of samples.

An asymptotic form for the P-value can now be obtained in the limit of large ν using Eqns. (58) and (68). Defining $t^2/\nu = \bar{a}^2/s^2 \equiv t_0^2$ and using $M =$

2 in Eqn. (68) leads to the asymptotic form of the **two-sided** t-distribution

$$Q(t|\nu) \to I_{\frac{1}{1+t_0^2}}\left(\frac{N_{\rm dof}}{2}, \frac{1}{2}\right) \sim \frac{\Gamma\left(\frac{N_{\rm dof}+1}{2}\right)}{\Gamma\left(\frac{N_{\rm dof}}{2}+1\right)\,\Gamma\left(\frac{1}{2}\right)} \frac{(1+t_0^2)^{\frac{-(N_{\rm dof}-1)}{2}}}{t_0}$$

$$\sim (1+t_0^2)^{-\frac{N_{\rm dof}}{2}} \sqrt{\frac{2(1+t_0^2)}{\pi\, N_{\rm dof}\, t_0^2}}\,, \tag{70}$$

which works fairly well for our cases if $t > 3$. Ignoring the ν-dependence of t would have led to a different asymptotic form.

VIII. Analysis of Fractional Residuals

We first explore the consequences of writing each fractional residual as a function of the random error $\hat{\epsilon}_i$ and at the same time expand $F(x_i, \{\bar{a}_M\})$ about the (exact) points $\{\mathbf{a}_M\}$ using Eqns.(24) and (26)

$$\hat{r}_i = \frac{\mathbf{y}_i - F(x_i, \{\bar{a}_M\}) + \hat{\epsilon}_i}{\sigma_i} \longrightarrow \frac{\hat{\epsilon}_i - d_i^M A^{-1}_{MN} \hat{E}_N}{\sigma_i}\,. \tag{71}$$

Note that $\overline{\hat{\epsilon}_i} = 0$ implies $\overline{\hat{r}_i} = 0$, but $\overline{\hat{\epsilon}_i} \neq 0$ implies $\overline{\hat{r}_i} \neq 0$, unless there is an accidental cancellation between the direct and correlation terms. Calculating the residuals correlation $\overline{\hat{r}_i \hat{r}_j}$ leads to the correlation function

$$\overline{\hat{r}_i \hat{r}_j} = \delta_{ij} - \frac{d_i^M\, A^{-1}_{MN}\, d_j^N}{\sigma_i \sigma_j}\,. \tag{72}$$

Setting $i = j$ and summing over i verifies that $\sum_{i=1}^{N} \overline{\hat{r}_i^2} = N - M$, as we found in Eqns. (22) and (28). The second term in (72) expresses the M correlations in the residues that result from fitting the M parameters to the data.

The mean $\bar{r}$ and variance σ^2 of the N (fractional) residuals are easy to compute. Our residuals need not have vanishing mean, as noted above. If these residuals are collected and binned, we can construct their (binned) probability distribution. For this procedure to have any significance, however, the fractional residuals should be roughly homoscedastic (i.e., homogeneous variance) in the variable x. In our case there is no reason to believe that our residuals correspond exactly to a Gaussian distribution, but they might

be a reasonable approximation to one. We therefore **assume** a Gaussian (probability) distribution normalized to N residuals

$$p(r) = \frac{N}{\sqrt{2\,\pi}\;\sigma}\exp\left[-\frac{(r-\bar{r})^2}{2\,\sigma^2}\right], \tag{73}$$

for the set of fractional residuals $r = \{r_i\}$ from the completed fit, recalling from Eqn. (32) that $r_i = \frac{y_i - F(x_i,\{\bar{a}_M\})}{\sigma_i}$. We next bin a Gaussian with the calculated residual mean, $\bar{r}$, and variance σ^2. If the βth (residuals) bin has lower breakpoint, $r_{\beta-1}$, and upper breakpoint, r_β (with a corresponding bin width, $\delta_\beta = r_\beta - r_{\beta-1}$), the expected value for the βth bin of the Gaussian is

$$G^{\beta}_{\text{bin}} = \frac{N}{2}\left(erf\left(\frac{r_\beta - \bar{r}}{\sqrt{2}\,\sigma}\right) - erf\left(\frac{r_{\beta-1} - \bar{r}}{\sqrt{2}\,\sigma}\right)\right), \tag{74}$$

where

$$erf(z) = \frac{2}{\sqrt{\pi}}\int_0^z e^{-t^2}dt\,. \tag{75}$$

Note that if δ_β is much less than other length scales, we can approximate: $G^{\beta}_{\text{bin}} \approx \delta_\beta\; p(r_\beta)$, as expected.

Inspection of binned residuals and binned Gaussian distributions shows that they are in reasonable agreement. The primary problem is that outliers (more than $3\,\sigma$ from the fit) have a large impact on the variance of the residuals by inflating the tail of the distribution of residuals, while every Gaussian has a very minimal tail in that regime.

In an effort to be more quantitative about the agreement between the binned residuals and the binned (**assumed**) Gaussian probability distribution, we resort to Pearson's X^2 test (we use the upper-case X^2 here rather than the lower-case χ^2 in order to distinguish it from the many other quantities denoted by the latter, including Eqns. (3) and (4)). In this procedure we first compute a test statistic, and then use the test statistic to compute the probability that the assumed Gaussian form is consistent or inconsistent with the binned data, based on an **arbitrary** cutoff probability that is typically taken to be 0.05. That is, if the test statistic leads to a probability of 5% or less, the hypothesis that the underlying distribution is Gaussian can be rejected. Values greater than 5% show consistency only, not proof of validity. The 5% cutoff is common but not universal. Note again that one is testing the *hypothesis* that a Gaussian is the correct distribution, and in

effect computing the probability that statistical fluctuations can cause the difference between the observed and expected values in the various bins. If the probability is too small, we can choose to reject the hypothesis. Note that we expect the results to be bin-size dependent, since smaller numbers of residuals in each bin mean larger fluctuations are possible, and this will improve the statistic.

Pearson's test statistic for n_b bins is given by (see Hoel [1])

$$X^2 = \sum_{i=1}^{n_b} \frac{(O_i - E_i)^2}{E_i} \, , \tag{76}$$

where O_i and E_i are the observed and expected frequencies in bin i, and $\nu = n_b - n_c$ is the number of degrees of freedom, where n_c is the number of constraints (i.e., combinations of data) used in constructing the Gaussian. In our case the latter is always 3, corresponding to the total number of points N, the mean $\bar{r}$, and the variance σ^2.

For large N the probability distribution used for comparison is the standard χ^2 distribution previously defined in Eqns. (51)-(53). Thus if "P" $\equiv Q(n_b - 3, X^2) < 0.05$, the binned data are very likely NOT represented by a Gaussian distribution. This number or "P-value" is conventionally denoted by "P", which hopefully will not lead to confusion with the notation for distribution functions previously defined.

One caveat is that the X^2 distribution is only an approximation to another discrete distribution function (e.g., a multinomial distribution) and that the number in any one bin must be (empirically), $E_i \geq 5$ (see Hoel [1]). Small bin numbers occur only in the far tails of our histograms, so that it is necessary to check if any bins are smaller than that. If this occurs one combines the outer bins until the composite bin has size ≥ 5.

The Pearson test statistic is weighted so the small parts of the distribution function can be important. The tails of the Gaussian therefore carry a substantial weight and problems with outliers can and do lead to a rejection of the Gaussian hypothesis. Even though our Bacteria residuals "look good" when plotted together with a Gaussian, they do not score well using Pearson's test. On the other hand a "reduced data set" (viz., after **arbitrarily** removing outliers $>$ 3 s.d.) does much better. Binning the reduced set into 9 bins leads to $P = 0.025$ (a failure), while 14 bins produces $P = 0.075$ (a success). Clearly the test is not independent of bin size, but indicates that apart from outliers our Bacteria residuals conform fairly well to a Gaussian

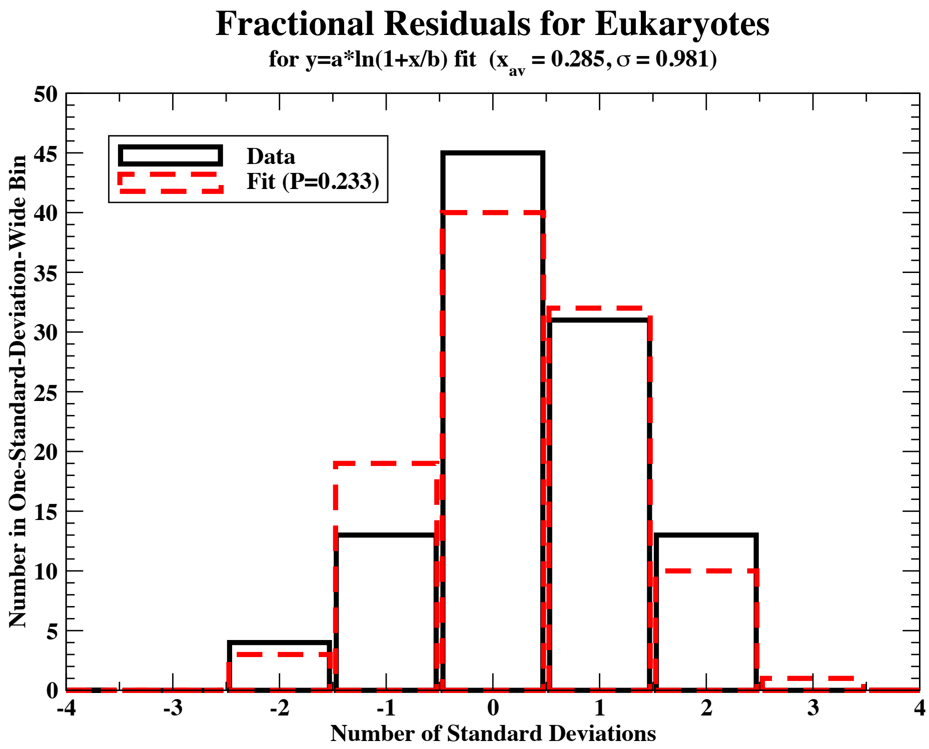

Figure 3: **Binned Eukaryota Fractional Residuals.** Genome-wide fractional residuals (in black) sorted into 7 one-standard-deviation-wide bins compared to an assumed Gaussian distribution with the same mean and variance (in red).

distribution. The Eukaryota fare much better and achieve a decent Pearson score, but one that is also bin-size dependent. The example in Fig. (3) uses 7 bins and has $P = 0.233$. Increasing the number of bins successively to 9, 14, and 27 produces $P = 0.549$, 0.525, and 0.544, respectively. Note that no "reduced data set" was **ever** used for our fits, except for the diagnostic discussed just above. We had to live with the outliers, since we did not understand their origin.

Finally, we note again that the distribution of residuals about the best-fit line will in general be asymmetric. In Figure (1) of the paper there are obviously more data points above the fitted Eukaryota line than below. In quantitative terms we show below that the distribution of points above and below the fit are rather accurately determined by the mean value $\bar{r}$ of the fractional residuals distribution. For simplicity we first assume that that distribution is given approximately by the product of a symmetric distribution ($P_0(r) = P_0(-r)$) with a small asymmetry-generating factor determined by a dimensionless parameter s

$$P_{\mathrm{f-r}}(r) = P_0(r)\left(1 + \frac{s\,r}{\sigma}\right), \tag{77}$$

where $\int_{-\infty}^{\infty} dr\ P_0(r) = 1$, $\int_{-\infty}^{\infty} dr\ r\ P_0(r) = 0$, and $\int_{-\infty}^{\infty} dr\ r^2\ P_0(r) = \sigma^2$ (the variance of P_0). We determine the value of $\bar{r}$ using Eqn. (77)

$$\bar{r} = \int_{-\infty}^{\infty} dr\ r\ P_{\mathrm{f-r}}(r) = \frac{s}{\sigma}\int_{-\infty}^{\infty} dr\ r^2\ P_0(r) = s\,\sigma\,. \tag{78}$$

The fractions of data points above and below the fit line are given by

$$n_+ = \int_0^{\infty} dr\ P_{\mathrm{f-r}}(r) = \frac{1}{2} + \frac{s}{\sigma}\int_0^{\infty} dr\ r\ P_0(r) \tag{79}$$

and

$$n_- = \int_{-\infty}^{0} dr\ P_{\mathrm{f-r}}(r) = \frac{1}{2} - \frac{s}{\sigma}\int_0^{\infty} dr\ r\ P_0(r)\,. \tag{80}$$

We use a normalized Gaussian to estimate the integral in Eqns. (79) and (80)

$$P_0(r) = \frac{1}{\sqrt{2\,\pi}\,\sigma}\exp\left[-\frac{r^2}{2\,\sigma^2}\right], \tag{81}$$

and find that the integral equals $\sigma/\sqrt{2\pi}$. Thus we have

$$n_\pm = \frac{1}{2} \pm \frac{\bar{r}}{\sigma}\frac{1}{\sqrt{2\pi}} = \frac{1}{2} \pm \frac{s}{\sqrt{2\pi}}\,. \tag{82}$$

Our Eukaryota fits have $\bar{r}/\sigma = 0.285$ or a predicted value of

$$n_{\pm}^{\text{Euk}} = \begin{cases} 0.61 \\ 0.39 \end{cases} \tag{83}$$

compared to actual values, $n_+ = 0.60$ and $n_- = 0.40$, which provides excellent agreement. Our Bacteria fits have $\bar{r}/\sigma = 0.106$, which leads to

$$n_{\pm}^{\text{Bac}} = \begin{cases} 0.54 \\ 0.46 \end{cases} \tag{84}$$

compared to actual values, $n_+ = 0.54$ and $n_- = 0.46$, which are also in excellent agreement with the prediction.

Figure (4) provides an alternative explanation for the number discrepancy above and below the Eukaryota fit line. The slight shift from the black fit line to the $\bar{r}/\sigma$ line moves about 10% of the upper points into the lower category, making them about equal in numbers. This demonstrates in a different way the role that a non-vanishing $\bar{r}$ has on the distribution of points above and below the fit line.

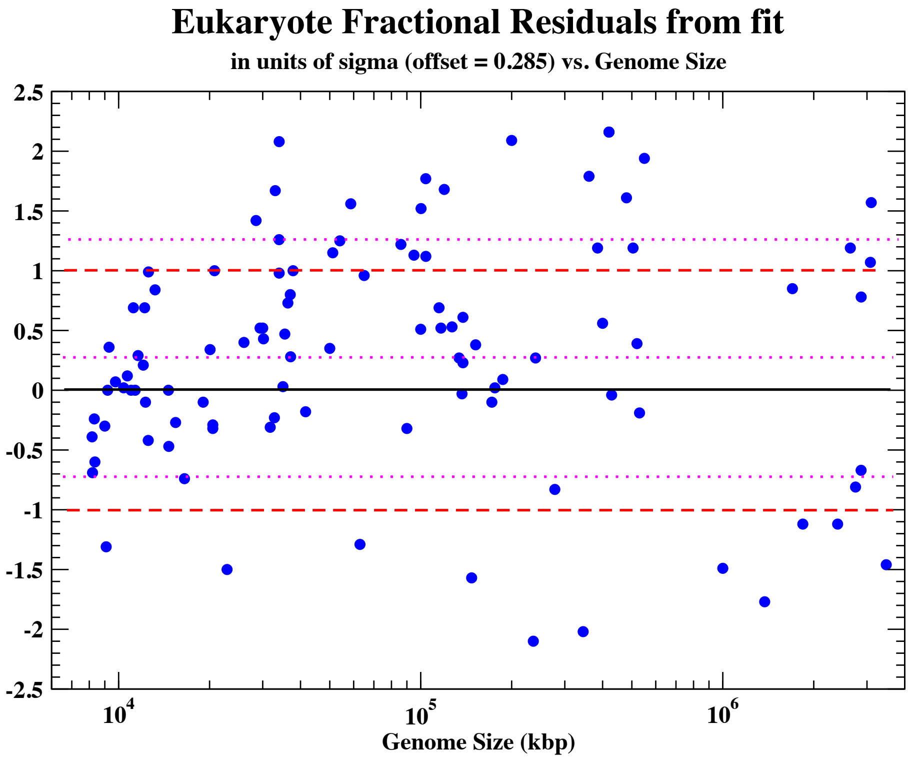

Figure 4: **Eukaryota Fractional Residuals.** Fractional residuals as a function of genome size in kbp. The black line corresponds to the fit, while the red dashed lines are one standard deviation away. The dotted magenta line at 0.285 is $\bar{r}/\sigma$, while the other two dotted magenta lines are one standard deviation away from $\bar{r}/\sigma$.

References

[1] Paul G. Hoel, *Introduction to Mathematical Statistics*, (Wiley, New York, 1958).

[2] W. H. Press, B. P. Flannery, S. A. Teukolsky, and W. T. Vetterling, *Numerical Recipes*, (Cambridge Univ. Press, 1986). See especially p. 503.

[3] Daniel S. Wilks, *Statistical Methods in the Atmospheric Sciences*, (Academic Press, San Diego, 1995), pp. 163-169.

[4] L. Lopez and J. Sesma, *Integral Transforms and Special Functions* **8**, 233 (1999).
